# An analysis of suicide trends in Scotland 1950–2014: comparison with England & Wales

**DOI:** 10.1186/s12889-017-4956-6

**Published:** 2017-12-20

**Authors:** Nadine Dougall, Cameron Stark, Tim Agnew, Rob Henderson, Margaret Maxwell, Paul Lambert

**Affiliations:** 1 School of Health & Social Care, Sighthill Campus, Edinburgh Napier University, Edinburgh, EH11 4BN UK; 2 Department of Public Health, NHS Highland, Assynt House, Beechwood Park, Inverness, IV2 3BW UK; 3 NMAHP Research Unit, Faculty of Health Sciences & Sport, University of Stirling, Stirling, FK9 4NF UK; 4 School of Applied Social Science, Colin Bell Building, University of Stirling, Stirling, FK9 4LA UK

**Keywords:** Scotland, England, Wales, UK, Suicide, Age period cohort analysis, Epidemiology, Deaths of undetermined intent, Deaths of intentional self-harm

## Abstract

**Background:**

Scotland has disproportionately high rates of suicide compared with England. An analysis of trends may help reveal whether rates appear driven more by birth cohort, period or age. A ‘birth cohort effect’ for England & Wales has been previously reported by Gunnell et al. (B J Psych 182:164-70, 2003). This study replicates this analysis for Scotland, makes comparisons between the countries, and provides information on ‘vulnerable’ cohorts.

**Methods:**

Suicide and corresponding general population data were obtained from the National Records of Scotland, 1950 to 2014. Age and gender specific mortality rates were estimated. Age, period and cohort patterns were explored graphically by trend analysis.

**Results:**

A pattern was found whereby successive male birth cohorts born after 1940 experienced higher suicide rates, in increasingly younger age groups, echoing findings reported for England & Wales.

Young men (aged 20-39) were found to have a marked and statistically significant increase in suicide between those in the 1960 and 1965 birth cohorts. The 1965 cohort peaked in suicide rate aged 35-39, and the subsequent 1970 cohort peaked even younger, aged 25-29; it is possible that these 1965 and 1970 cohorts are at greater mass vulnerability to suicide than earlier cohorts. This was reflected in data for England & Wales, but to a lesser extent.

Suicide rates associated with male birth cohorts subsequent to 1975 were less severe, and not statistically significantly different from earlier cohorts, suggestive of an amelioration of any possible influential ‘cohort’ effect.

Scottish female suicide rates for all age groups converged and stabilised over time. Women have not been as affected as men, with less variation in patterns by different birth cohorts and with a much less convincing corresponding pattern suggestive of a ‘cohort’ effect.

**Conclusions:**

Trend analysis is useful in identifying ‘vulnerable’ cohorts, providing opportunities to develop suicide prevention strategies addressing these cohorts as they age.

**Electronic supplementary material:**

The online version of this article (10.1186/s12889-017-4956-6) contains supplementary material, which is available to authorized users.

## Background

Suicide is a global public health issue with an estimated 804,000 people dying worldwide in 2012, corresponding to a standardised mortality rate (SMR) of 11.4 per 100,000 [1]. It is one of the leading causes of death in younger people (aged 15-44) and rates have increased by 60% in some countries during the last half-century [2]. In keeping with this alarming global picture, Scotland, as a constituent nation of the UK declared suicide to be a significant public health concern, prompting the Scottish Government to set a target to reduce the suicide rate in Scotland by 20% over a decade to 2013. This target was largely achieved with an overall 19% reduction to 2013 and an estimated 746 deaths, equating to a Standardised Mortality Rate (SMR) of 13.5 per 100,000 [3].

Although the suicide mortality rate in Scotland is analogous to that observed internationally and rates have reduced somewhat over the last decade, there remains substantial scope for further reductions to be achieved. It is *not* widely known that the Scottish rate remains disproportionately higher than that of its adjoining neighbour, England, and especially so for men – in 2008 the male suicide rate for Scotland was 24.1 per 100,000 of the population, almost double that of England at 12.6 per 100,000 [4]. This disproportionate impact of suicide on Scotland is even more notable in a comparison of younger men (15-44 years); whilst Scottish suicide rates for this group remained about double that of England, the gap widened between SMRs for the period 1998-2004, and were much more elevated at 36.9 and 19.1, respectively [5].

Considering national comparison data for more recent years of data available, the contrast between these two neighbouring countries remains marked. The standardised suicide rate among working age Scottish men was estimated for 2010 to be 73% higher than that found for England & Wales (denoting a combined dataset of both England & Wales both here and in subsequent mentions), whilst the corresponding Scottish female rate was almost double that for England & Wales [6]. Epidemiological studies of temporal trends in suicide have reported that for Scotland, rates increased in the last three decades of the twentieth century followed by a suggested downward trend over the last decade to 2010 [7, 8]. England & Wales on the other hand observed a similar increase in suicide rates between 1975 and 1990, followed by a decrease in rates a decade earlier than Scotland for males and females [9] which has persisted to the present day [10].

A recent cross-national comparison of longitudinal trends concluded there was a marked divergence from 1992 to 2008, with Scottish suicide rates having markedly increased rates relative to those decreased rates observed for England & Wales [8]. However, in summarising overall trends the underlying contributions from different age groups is masked. For instance, in the 1990s England & Wales experienced decreases in the male suicide rate for all age groups except for a relative increase in the 25-34 year old age group [11, 12]. This differs from the picture in Scotland for the 1990s when increases in *all* age-groups up to 44 years old were experienced, with corresponding decreases observed in groups 45 years and over [8, 13, 14]. Finding candidate factors which help explain these cross-national UK differences is of interest to enable more effective targeting of suicide prevention strategies.

An analysis of trends is one approach which can help tease out whether rates observed are more consistent with patterns that reflect differences between the decade people were born (cohort effect) or died (period effect) or of the changing condition of age. Consideration of age, period and cohort patterns in turn can lead to a better understanding of different influences in time, and may yield predictive information on future time periods where some populations may be more at risk. Age effects refer to differences in rates observed as a consequence of the impact of ageing on people within the same cohort. Period effects refer to patterns associated with a particular year or decade, such as the increase in deaths in the late 1920’s and early 1930’s at the time of the Great Depression, or the increase in deaths related to coal gas [15]. Cohort effects reflect long term impact of a generation’s exposure to particular conditions, such as a lack of work as they entered the work force. If birth cohorts carry with them an increased (or decreased) susceptibility to suicide throughout their lives then this would have important implications for the targeting of suicide prevention efforts.

From a statistical perspective, there are ongoing debates about plausible strategies for disentangling age, period and cohort effects, as the linear dependency of all three time-dependent measures mean that inferences from this sort of analysis are highly problematic [16–21]. For instance, an effect that appears to be a cohort effect may well be a combination of age and period effects. One approach is to explore trends via graphical analyses, and interpret trends observed based on some assumptions with a reasonable rationale. Assumptions can be based on pre-existing research evidence, for example, known discrete period effects. This strategy was adopted in an earlier ‘cohort analysis’ presented by Gunnell et al. for data from England & Wales [12]; those deaths by overdose and gassing were excluded from the full dataset, changing the birth cohort patterns and supporting the suggestion that popularity and lethality of particular methods of suicide have a period effect.

Gunnell et al. (2003) reported that an increase in suicide rates in successive birth cohorts was evident from 1940 onwards, and in males in particular, concluding that an increase in overall male suicide rates is inevitable if this trend continues into middle and older age [12]. A comparison ‘cohort analysis’ of Scottish data has not been reported, and is now timely as recent national data points to an evolving picture of a relatively increased suicide rate in middle-aged men aged 35 to 54, compared with a relatively decreased rate in those less than 34 years [22].

Therefore the aim of this study was to use Scotland’s suicide data to replicate the reported analysis for England & Wales by Gunnell et al. (2003), to highlight any similar potential cohort patterns within Scotland, and ascertain any cross-national differences in a direct comparison.

## Methods

### Study data

Published suicide information from the vital event reports of the National Records of Scotland (NRS) was used for this study. These reports are published annually, and include summarised information on causes of death by age group and gender. These data are openly available, do not require permission to use, and data from 1974 onwards is accessible online to download. This study spanned sixty five years from 1950 to 2014, with corresponding codes defining deaths by suicide from various iterations of the International Classification of Diseases (Table 1). As more historical data from 1950 to 1973 were not available via online access, two authors (TA and RH) manually extracted the data from reports held at the NRS, and entered both these and the electronic data for more recent years on to an Excel spreadsheet. Corresponding annual general population estimates by age and gender were also obtained from NRS. A category for deaths which were undetermined as to whether the death was accidentally or purposely inflicted was introduced in 1968 with ICD8 and its corresponding codes (Table 1), therefore categories of deaths as a result of intentional self-harm and of undetermined intent were available for analysis.

**Table 1:** ICD catalogues and codes

Years	ICD catalogue and codes for ‘Intentional self-harm’	ICD catalogue and codes for ‘Deaths of undetermined intent’
1950 to 1957	ICD6 E970-E979	Did not exist
1958to 1967	ICD7 E970-E979	Did not exist
1968 to 1978	ICD8 E950-E959	ICD8 E980-E989
1979 to 1999	ICD9 E950-E959	ICD9 E980-E989
2000 to 2014	ICD10 X60-X84, Y87.0	ICD10 Y10-34, Y87.2

ICD catalogue and codes used for deaths by suicide by the National Records of Scotland during the timespan 1950-2014

### Cohort analysis

We were interested in exploring long-term temporal trends in suicide outcome from 1950 to 2014 and any patterns suggestive of effects of age, period (year of death) and cohort (year of birth). We describe this descriptive analysis of trends as ‘cohort analysis’ to keep the terminology consistent with the work of Gunnell et al. (2003) although it is recognised that an analysis of trends is a more accurate description.

There is a linear interdependency between the variables age, period and cohort and if they are all in the same functional form it is impossible to distinctively identify patterns associated with all three variables, known as the identification problem [15, 21]. Put simply, this can be expressed as follows:

Age = Period – Cohort.

Period = Cohort + Age.

Cohort = Period - Age.

It is possible based on prior evidence to make some assumptions which can help facilitate data interpretation. Studies have previously reported on discrete effects of period on suicide, although mechanisms such as diffusion mean that the empirical impact of period effects may be more gradational in character. For instance, suicide intervention strategies could have discrete period effects with some enduring legacy effects remaining from previous interventions, and lag effects from implementation to measurable impact. Accordingly, a pattern which persists for many decades is much less likely to be a completely continuous period effect, and more likely a consequence of successive cohorts. Our analysis seeks to emulate this previous analysis reported for England & Wales by Gunnell et al. [12] which explored cohort patterns by adopting high level descriptive summary and graphical analysis. We repeated the graph formats produced earlier [12] to provide a comparison with England & Wales data.

Age and gender specific suicide rates per 100,000 of the Scottish population from 1950 onwards were derived for every year by dividing suicide numbers for each five year age band (15-19, 20-24,…,80-84, 85+) by the corresponding age and gender matched general population estimates. Although the use of age specific suicide rates does not help overcome the linear relationship between age, period and cohort variables, they do enable valid comparisons to be made between gender and calendar time, and differences in country patterns.

Suicide completers were classified into six groups based on age at year of death aged 15-24, 25-34, 35-44, 45-54, 55-64 and an older group of 65+ years. We aggregated age at death into ten year age bands to simplify the graphical representation. The gender specific mean ages in five year death periods were then estimated and plotted on moving average charts to examine the relationship between incidence of suicide and the age of suicide completers during each death period. Each five year moving average point was plotted on the mid-year point, e.g. data for period 1950-54 was plotted on 1952, meaning that each five year period has a ‘lag’ period of 2.5 years.

We investigated the cohort patterns by plotting suicide rates in age bands, summarised by weighted mean rate within each 5 year death period to 2014. As each five year death period consisted of five possible ages at death, the birth cohort consisted of people born over a possible range of nine years. For example, for the age band 25-29 years with deaths data for the period 1960-1964, the possible birth range corresponded to the nine year period 1941 to 1949. To plot the graphs we assigned the middle year in which each nine year birth range centred on as the notional birth cohort year, therefore 1941-1949 had 1945 as the birth cohort year; this follows the method previously described in more detail by Gunnell et al. [12]. Where relevant, 95% Confidence Intervals (95% CI) using ‘Mid-P’ exact confidence limits for age-specific rates were estimated using the ‘Quickcalc’ tab of ‘Episheet’ [23], available online (personal communication with Prof Ken Rothman).

## Results

### Male patterns by age and period

**Fig. 1:**
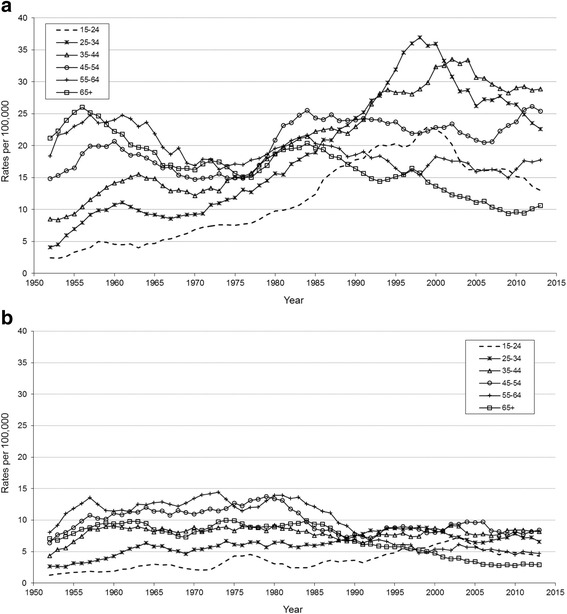
Age specific suicide rates in Scotland for (**a**) men and (**b**) women. 1950-2014 (5 year moving averages) in ten year age bands for suicide as a result of intentional self-harm only (undetermined intent deaths excluded)

The age and period graph (Fig. 1a) suggests a pattern reflecting some change over the period in how suicide rates are distributed by age groups. The suicide rates of the younger age groups increased markedly over time; those aged 25-34 and 35-44 reaching the highest rates of any group in the period around 2000, before decreasing to 2014. In contrast, those aged 45-64 have seen increasing suicide rates for much of the last decade and have now reached similar rates as these younger men. Rates for older age groups tended to be more stable through the period, but are characterised by steady decline through the period for the oldest group (65+). Post WW2, the burden of suicide was highest in those middle and older groups of men (>44 years).

### Female patterns by age and period

The contrast for women compared with their male counterparts is striking (Fig. 1a & b). From the 1990s onwards the disproportionate burden of suicide is seen for men, and in particular younger men aged 25-44. Although the successive women in younger age-groups experienced a steady increase during this same period, this increase was much more modest and the most recent data suggest this trend has now reversed or stabilised (Fig. 1b).

Conversely, trends for older women can be seen to have been highest in the 1950s and remained fairly constant until the 1980s with a stable age group ordering. From the 1980s the older women’s rates consistently decreased to become the lowest ever in most recent years, with all age groups demonstrating convergence. In short, although variations in age group orderings for women are also seen during the 1990s, women were not affected by suicide to the same degree as men.

A feature of ‘moving average’ charts (Fig. 1a & b) is that individual data points have been ‘smoothed’ to permit trends to stand out. One limitation here is that the female averaged patterns are derived from far fewer individual observations (i.e. increased variability in the mean of the distribution of values, not the distribution of values itself) and it is unlikely there are statistically different differences for the female age groups.

### Male patterns by age and period - comparison with England & Wales

For men, there are striking differences observed between Scotland, and England & Wales (see Fig. 1a, and ‘Additional file 1: Figure S1’ reproduced from Gunnell et al. [12]). The 1950s age-specific suicide rates in England & Wales were almost twice as high as those in Scotland and followed a more exaggerated pattern with steeper declines to the 1970s observed. The underlying suicide pattern between the countries was not dissimilar from the 1950 to mid-1970s in that there was consistent and identical age group ordering, with rates which steadily decreased and converged over time till the nations were broadly similar.

From the mid-1970s to 2000 a markedly divergent pattern emerged between nations; the Scottish suicide rate increased dramatically for men aged 25-44, reaching rates which were disproportionate and at least double that of England & Wales. Rates in men aged 25-44 also rose in England & Wales but they never exceeded those of the other age groups (see Fig. 1a, and ‘Additional file 1: Figure S1’ reproduced from Gunnell et al. [12]).

**Fig. 2:**
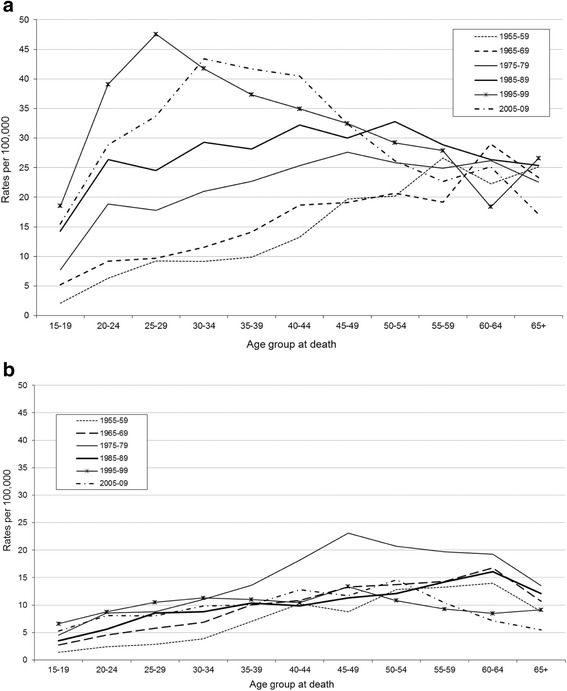
Age and period patterns in Scotland for (**a**) men and (**b**) women. Suicide as a result of intentional self-harm and deaths of undetermined intent

Age and period rates for Scottish males were plotted in a different format, following Gunnell et al. [12], (Fig. 2a). This figure features a pattern of stable ordering of suicide rates, (with the exception of those >49 years) up to the 1990s, when the pattern is disrupted, indicative of a possible period influence in these younger groups but only until the 1990s. As in Scotland, the combined territory of England & Wales also had subsequent five year death periods spanning 1955-1999 which featured parallel shifts upwards in suicide rates for each passing decade for younger age groups up to the 1990s (see Fig. 2a; and ‘Additional file 2: Figure S2’ reproduced from Gunnell et al. [12]). We can conclude that for both Scotland, and England & Wales, these patterns by period were very similar for men <35 years (with increased suicide rates with each passing decade to the end of the last century), but quite different for those men older than 35.

### Female patterns by age and period - comparison with England & Wales

The suicide rates for older women in England & Wales were almost double that seen in Scotland in the 1950-60s, but decreased with every decade to 2000, dropping below the Scottish equivalent rates, and converging on a stable rate for all age groups, (see Fig. 1b, and ‘Additional file 1: Figure S1b’ reproduced from Gunnell [12]). Overall the suicide trend for women in Scotland was more stable with fewer fluctuating features. Compared with England & Wales, although Scottish women historically completed suicide less frequently, from 1995 onwards the picture is reversed with higher female rates in Scotland. As female deaths by suicide are sparser than the male data more caution is required in drawing conclusions.

The corresponding female pattern for period (Fig. 2b) demonstrates a pattern broadly consistent across all age groups for every ‘period’ with the exception of the period 1975-79 which has higher suicide rates in Scotland for those women between 40 and 59 years. In comparison, for England & Wales women >45 years show a consistent decrease with every subsequent decade to 1999, (see ‘Additional file 2: Figure S2b reproduced from Gunnell [12]).

### Male patterns by age and cohort

**Fig. 3:**
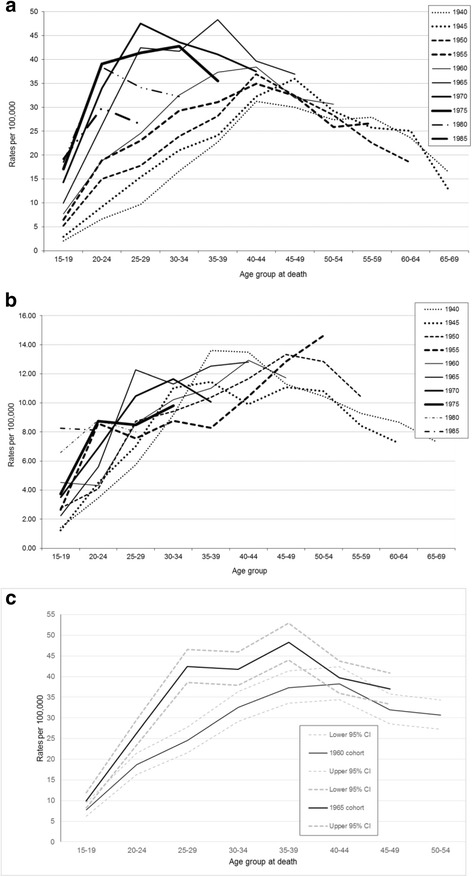
Birth cohort patterns in Scotland for (**a**) men, (**b**) women, and (**c**) 1960 and 1965 male cohort trajectories with 95% Confidence Intervals. Suicide as a result of intentional self-harm and deaths of undetermined intent for cohorts 1940 to 1985

Figure 3a and b set out suicide rates by birth cohorts for Scottish men and women, respectively. For men it can be seen that all cohorts have suicide rate trajectories which peak at higher numbers and in younger age groups with each successive cohort. In comparison with the 1960 cohort, there is a marked stepped change in worsening trajectories with the 1965 and 1970 cohorts. The 1965 cohort peaked in suicide rates for 35-39 year olds in 2000-2004, and the 1970 cohort peaked even younger for 25-29 year olds in 1995-1999. To be clear, Fig. 3a uses the same underlying data as Fig. 1a but presented in a different way, and these conclusions on cohort are made using Fig. 3a whilst ignoring the conclusions from Figs. 1a and 2a.

The trajectories for the 1960 and 1965 cohorts alongside their respective 95% CIs demonstrate a pattern consistent with a statistically significant increase between these cohorts for those aged 20-24 to 35-39 (Fig. 3c). Although this statistically significant difference is observed, it is not known whether this is a consequence of cohort or a combination of the factors of age, period or cohort.

### Female patterns by age and cohort

For women the pattern seen for men is not so apparent (Fig. 3b). The marked stepped change in increased suicide rates with the 1965 and 1970 cohorts is not obvious, and there does not appear to be a convincing greater risk of suicide as these particular cohorts aged. Aside from the lack of a pattern suggestive of a convincing female birth cohort effect, presenting the data in this way (Fig. 3b) does not add to what has been expressed above – older cohorts (and therefore older age groups as seen when considering Fig. 1b on its own) have higher suicide rates, and more recent cohorts are at risk of dying younger, but in fewer numbers.

### Male patterns by age and cohort - comparison with England & Wales

The cohort patterns for both Scotland, and England & Wales are broadly similar but differences are more marked in Scotland. For both countries, suicide rates in each successive birth cohort peaked in earlier age groups, and in higher numbers for more recent cohorts (see Fig. 3a, and ‘Additional file 3: Figure S3a’ reproduced from Gunnell [12]). Gunnell et al. concluded that “successive male birth cohorts born after 1940 carried with them, as they aged, a greater risk of suicide than their predecessors, although this effect diminished for 1975 and 1980 cohorts”.

The cohort pattern for England & Wales also suggests that this “greater risk of suicide” is different in those born from 1965 onwards with increased suicide rates in younger people. The Scottish data features this same difference but with a disproportionate additional impact of suicide on the 1965 and 1970 cohorts, with those aged 35-39 years and 25-29 years during the 1990s dying in greatest numbers, respectively (Fig. 3a, and ignoring conclusions from other graphs as each graph leads to different conclusions because of the APC identification problem).

## Discussion

### Main findings

Scottish male cohort trajectories demonstrated a pattern consistent with a statistically significant increase in suicide between 1960 and 1965 for those aged 20-39. The 1965 and 1970 cohorts peaked in suicide rate at age 35-39 and 25-29, respectively. It is possible that the 1965 and 1970 cohort are at greater mass vulnerability to suicide than earlier or subsequent cohorts, and that as they age there may yet be greater suicide rates in these cohorts as a consequence. Alternatively the period 1995-2004 may have posed increased risk to these cohorts; further research is needed to rule in or out explanatory discrete period effects of e.g. access to means of death which are recorded on death certificates and not available in this study for analysis. It is not possible to categorically establish if this pattern is one attributable to age, period or cohort, or a combination of all three factors.

One recent study has also now reported on this apparent cohort effect, and concluded that these cohort patterns were more pronounced for those living in the most deprived areas, and that the suicide rates in Scotland can be explained by a cohort effect consistent with exposure to neoliberal politics during the 1980s, an exposure experienced more in deprived communities [18].

### Temporal patterns of suicide in Scotland

In Scotland post WW2 years, the burden of suicide was highest on older men (>44 years) with rates decreasing to the 1980s, before stabilising thereafter. The inverse was seen for younger men (<45 years) who had the lowest suicide rates which steadily increased and peaked by the late 1990s, with this group continuing to bear the burden of suicide until 2000. From 2000 to 2014 decreasing rates are seen for those aged 15-34 alongside increasing rates for 45-54 year olds, leaving the greatest impact of suicide on those aged 35-54.

During 1998-2004, 17 of the top 20 local areas with the highest male suicide rate in the UK were located in Scotland, with the Shetland Islands, Eilean Siar, Highland and Glasgow City having the highest rates at 47.5, 44.1, 43.3 and 41.6 per 100,000 population, respectively [5]. Rurality may explain some patterns of excess suicide - for Highland, an excess of male deaths appeared to be associated with access to more lethal means and rural occupation [24, 25]; a relative dearth of contact with mental health services in the month prior to suicide was found for those that lived in ‘remote rural’ or ‘remote small towns’ [26]; enforced social isolation has also been proposed in a conceptual model of rural suicide [27]. However excess suicide has also been observed in some urban areas. An analysis of suicides in Greater Glasgow to 2001 concluded that the East end of Glasgow formed a large geographical cluster of young adult suicide which persisted for *two decades* and which was likely not explained by ‘contagion effects’, but rather more likely by a concentration of deprivation [28].

Although there are many age and period risk factor interactions at play, it is not possible to explain in simple terms what drove the patterns observed, but it is likely that regional differences fuelled by deprivation are partly to explain.

In contrast, female suicide rates for all age groups in Scotland have converged and stabilised in recent years with evidence that women have not been affected by the huge impact of suicide as seen for men. Although there was a pattern for women with a limited number of successive cohorts showing peaks in younger age groups with each generation, this was unclear and no firm conclusions could be made due to sparser data.

### Comparison with England & Wales

Temporal patterns of suicide for Scotland were markedly different to those in England & Wales, which had a much higher burden of suicide than Scotland in the 1950s. These patterns reversed over time so that Scotland became the country with disproportionate impact of suicide, compared to England & Wales. Men were affected more markedly than women over all years in both countries. In spite of the between country differences, the gender patterns within each country were similar, meaning gender is the bigger determinant with more overall predictive power.

The pattern reported for England & Wales by Gunnell et al. [12] of successive male birth cohorts from 1940 experiencing higher suicide rates peaking in younger age groups with each decade was also seen in Scotland. The differences in pattern with increased suicide rates for the 1965 and 1970 cohorts exist in both countries, but are more marked in Scotland.

Gunnell et al. explored the impact of discrete period events, and found that restricting access to lethal means (predominantly 1993 legislation on car exhaust emissions and the advent of catalytic converters) was effective in reducing suicide rates, i.e. when these period effects were controlled for, the pattern consistent with a cohort effect disappeared - there was no evidence that rates peaked earlier in later born cohorts (Additional file 3). We did not have access to the Scottish data by method of suicide and could not compare this discrete period effect. It has been reported elsewhere that deaths from motor vehicle exhaust fumes decreased in England alongside a corresponding increase in hanging deaths, whilst in Scotland hanging deaths were already increasing in men before deaths from motor vehicle exhaust fumes began to decline, with this increase being greater than the corresponding decrease [24]. Restricting access to means of suicide may have lasting impact in some countries, but for Scotland the discrete period effect of catalytic converter legislation did not achieve the same impact seen in England & Wales. Thus there may still be a greater risk in Scotland attributable to belonging to the 1965 and 1970 birth cohorts, for reasons which remain unclear.

This widening ‘suicide gap’ has been reported before, with the crossing over of increasing suicide rates in Scotland, and decreasing rates in England & Wales occurring in the 1960s, and differences since the 1990s being explained by a preference in the methods of hanging, suffocation or strangulation by young adult males [8].

It is plausible that regional differences in England & Wales data are obscured by using pooled national data, and that disaggregating these data would produce markedly different patterns. Analysis reported elsewhere concluded that between 1998 and 2004 large regional disparities existed between suicide rates in the countries of the UK and between local regions, and that deprivation as a risk factor fuelled these inequalities in suicide rates [5]. In 2011 the Office for National Statistics (ONS) published UK suicide data and estimated standardised male rates for 2011 ranging in England from 13.2 in London to 21.5 in North East England, with Wales being even higher at 22.5 [29]. Such regional variations have been noted within countries before and are typically associated with other risk factors [30]. Regional inequalities in suicide rates have also been characterised by markers of unemployment [31] and more recently, unemployment associated with the discrete period effect of the last UK recession [32, 33]; and low social integration, indicated by features of the proportion of single-person households, divorced people and population mobility [34].

A specific multilevel analysis exploring a range of factors between Scotland and England during 2001-2006, found that 57% of the excess suicide risk in Scotland was explained more by the area level measures of psychotropic drug prescriptions (proxy for poorer mental health), alcohol and drug misuse, with a relatively small contribution of deprivation and social fragmentation [35]. Therefore caution is required in reviewing national data patterns for men, and it is possible that Scottish data patterns observed may well find more concordance with specific regional English or Welsh patterns.

### Public health and future research implications

Although Scotland has made substantive progress in reducing suicide in recent years alongside national suicide prevention initiatives, it is not possible to know whether there is a direct causal link between such suicide prevention strategies and relative period decreases achieved from the early 2000s [36]. The first ‘Choose Life’ initiative was a ten year plan introduced in December 2002 aimed at reducing suicides by 20% by 2013 – however it appears the decline in suicides may have started before ‘Choose Life’ had a chance to have an impact and it is not possible to categorically know which particular age groups would have been impacted on most. These national initiatives coincided with a period of perceived economic expansion and lower unemployment until the 2008 UK recession.

Other comprehensive social changes were going on aside from economic fluctuations that may also have compelling plausible explanations for the suicide impact of the 1965 and 1970 male cohorts, such as the continuing impact of de-industrialisation, related unemployment and persistent effects of deprivation as previously mentioned. Major reforms in education with increasing attention to well-being and increased average time in education may also possibly have had a long term impact. The increased use of mobile technology and its impact on social connectedness may also need to be considered in explaining recent or future trends. Important legislative and political changes that have impacts for certain minority groups e.g. immigration, equality legislation will also have had their effects. Therefore, further research at the individual level (e.g. means of suicide especially drug overdose, occupation, educational attainment) on the 1965 and 1970 cohorts of males who were aged 25-39 at death may shed some light on suggested potentiality for risk in later life in the same birth cohorts. Such research should seek to identify risk factors or ‘exposures’ that lead to mass vulnerability of cohorts so as to minimise the longer term impact of such exposures, and in the planning of building resilience in future generations in the very early years when such ‘exposure’ risks re-appear.

### Strengths and limitations

One limitation of this comparison is that the data quality between countries is likely to differ, with potential variation in both coding and coding consistency across the years. We can also plausibly assume that data quality may be heterogeneous between age groups, with those younger age groups dying later having better quality data compared with their older counterparts dying a longer time ago. Graphical interpretations are reliant on the assumptions being made by each graph, and different assumptions would result in different interpretations. There was no matching data on specific suicide methods used, therefore it was not possible to compare the cohort trajectories using these different methods and our interpretation was guided by analyses reported elsewhere. In emulating the previous analysis for England & Wales by Gunnell et al. [12], the techniques used in this study were limited and the description of the methods expressed in terminology which takes account of known limitations. Nevertheless the utility of considering the factors of age, period and cohort in graphical terms has been demonstrated.

A strength of this study is the manual recovery of data prior to 1974 which permitted a long period of follow-up for the older cohorts. We were unable to do a comparison between the countries using a reference population given the historical data and the comparison with an earlier study; future research could standardise each country data to a standardised European population dataset to enable more robust comparisons to be made.

## Conclusions

This sort of trend analysis cannot confirm direct causal mechanisms of heightened suicide risk, but nevertheless can be helpful in identifying patterns which suggest opportunities to develop suicide prevention strategies specifically addressing cohorts and their mental health needs as they age. Male suicide rates peaked at higher numbers and in younger ages with each successive cohort. Specifically, there was a statistically significant increase in suicide rates for the 1965 birth cohort compared with the 1960 cohort, for men aged 20-39. The 1965 cohort peaked in suicide rates for 35-39 year olds in 2000-2004, and then the 1970 cohort peaked for 25-29 year olds in 1995-1999. There is no convincing evidence to suggest that the 1965 and 1970 cohorts may maintain increased risk for suicide as they age.

## Additional files

Additional file 1: Figure S1. Age-standardised suicide rates: 1950-1999 England and Wales (3-year moving averages) in (a) males and (b) females. (DOCX 135 kb)

Additional file 2: Figure S2. Suicide and undetermined death rates by time (of death) period in (a) males and (b) females. (DOCX 133 kb)

Additional file 3: Figure S3. Male rates of suicide and undetermined death in successive 5-year birth cohorts at different ages. (a) All suicides and undetermined deaths; (b) suicides and undetermined deaths except those by overdose and gassing; (c) overdose, gassing and undetermined deaths excluded. (DOCX 140 kb)

## Abbreviations

DUI: Deaths of undetermined intent
ICD: International Classification of Disease
ISH: Intentional self-harm
NHS: National Health Service
SMR: Standardised mortality rate
WW2: World War 2
