# Supplementary material for: An analysis of suicide trends in Scotland 1950–2014: comparison with England & Wales
Source: BMC Public Health. 2017 Dec 20;17:970. doi: 10.1186/s12889-017-4956-6 (PMC5738808; doi:10.1186/s12889-017-4956-6)
Supplement: Supplementary file 1 — Age-standardised suicide rates: 1950-1999 England and Wales (3-year moving averages) in (a) males and (b) females. (DOCX 135 kb) [file 12889_2017_4956_MOESM1_ESM.docx]

**Figure S1 Age-standardised suicide rates: 1950-1999 England and Wales (3-year moving averages) in (a) males and (b) females**
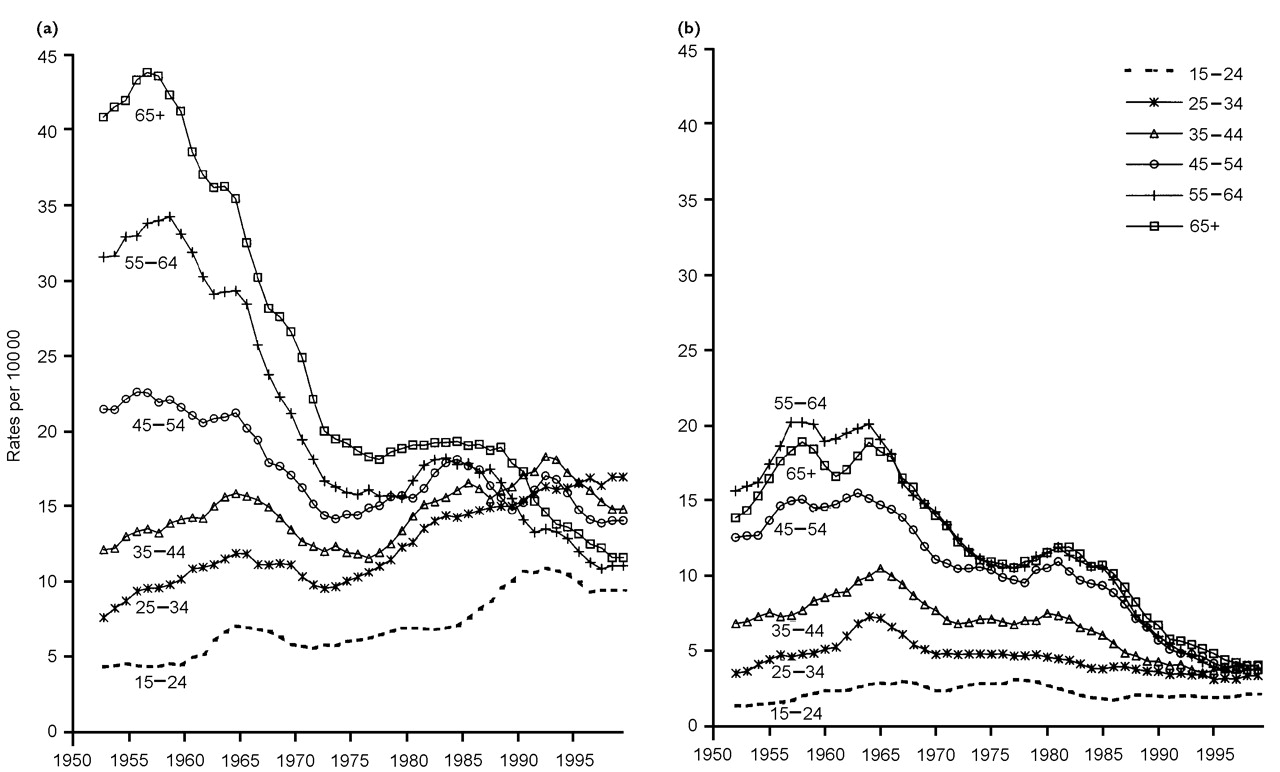


Reproduced with permission from Gunnell D, Middleton N, Whitley E, Dorling D, Frankel S: **Influence of cohort effects on patterns of suicide in England and Wales, 1950-1999***, B J Psych* 2003, **182**: 164-70
