# Supplementary material for: An analysis of suicide trends in Scotland 1950–2014: comparison with England & Wales
Source: BMC Public Health. 2017 Dec 20;17:970. doi: 10.1186/s12889-017-4956-6 (PMC5738808; doi:10.1186/s12889-017-4956-6)
Supplement: Supplementary file 3 — Male rates of suicide and undetermined death in successive 5-year birth cohorts at different ages. (a) All suicides and undetermined deaths; (b) suicides and undetermined deaths except those by overdose and gassing; (c) overdose, gassing and undetermined deaths excluded. (DOCX 140 kb) [file 12889_2017_4956_MOESM3_ESM.docx]

**Figure S3 Male rates of suicide and undetermined death in successive 5-year birth cohorts at different ages. (a) All suicides and undetermined deaths; (b) suicides and undetermined deaths except those by overdose and gassing; (c) overdose, gassing and undetermined deaths excluded.**


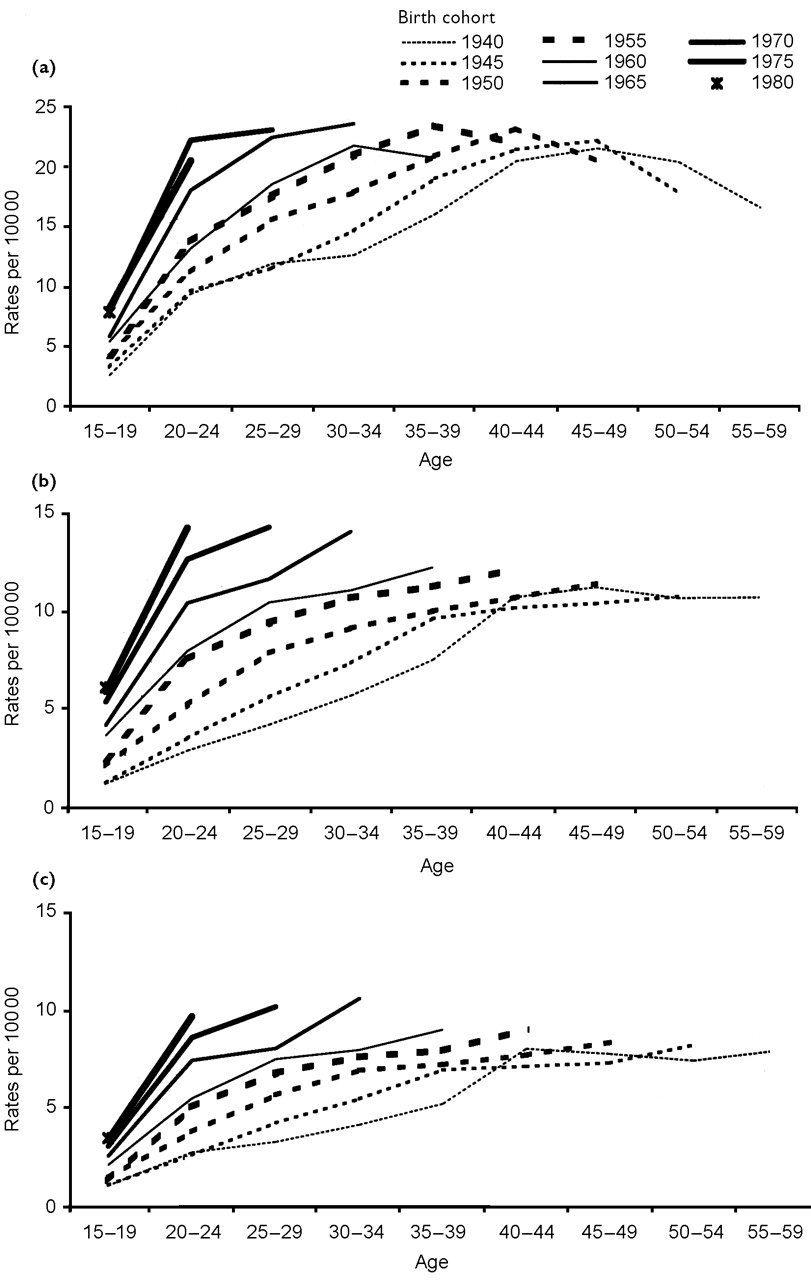


Reproduced with permission from Gunnell D, Middleton N, Whitley E, Dorling D, Frankel S: **Influence of cohort effects on patterns of suicide in England and Wales, 1950-1999***, B J Psych* 2003, **182**: 164-70
